# Predicting Major Depressive Disorder Using Neural Networks from Spectral Measures of EEG Data

**DOI:** 10.3390/bioengineering12111251

**Published:** 2025-11-16

**Authors:** Igor Kozulin, Ekaterina Merkulova, Vasiliy Savostyanov, Haonan Shi, Xinyi Wang, Andrey Bocharov, Alexander Savostyanov

**Affiliations:** 1 Faculty of Information Technologies and Mechanical-Mathematical Faculty, Novosibirsk State University, 630090 Novosibirsk, Russia; i.a.kozulin@yandex.ru (I.K.); k.shi@g.nsu.ru (H.S.); s.van14@g.nsu.ru (X.W.); 2 Institute of Computational Mathematics and Mathematical Geophysics SB RAS, 630090 Novosibirsk, Russia; 3 Scientific Research Institute of Neurosciences and Medicine, 630117 Novosibirsk, Russia; e.merkulova@g.nsu.ru (E.M.); vasilijsavostanov284@gmail.com (V.S.); bocharovav@neuronm.ru (A.B.)

**Keywords:** EEG, machine learning, artificial neural networks, major depressive disorder

## Abstract

Processing electroencephalogram (EEG) data using neural networks is becoming increasingly important in modern medicine. This study introduces the development of a neural network method using a combination of psychological questionnaire data and spectral characteristics of resting-state EEG. The data were collected from 71 individuals: 42 healthy and 29 with major depressive disorder (MDD). We evaluated four classes of algorithms—traditional machine learning, deep learning (LSTM), ablation analysis, and feature importance analysis—for two primary tasks: binary classification (healthy vs. MDD) and regression for predicting Beck Depression Inventory scores (BDI). Our results demonstrate that the superiority of a given method is task-dependent. For regression, an LSTM network applied to delta-rhythm EEG data achieved a breakthrough performance of R^2^ = 0.742 (MAE = 6.114), representing an 86% improvement over traditional Ridge regression. Ablation studies identified delta and alpha rhythms as the most informative neurophysiological biomarkers. Furthermore, feature importance analysis revealed a triad of dominant psychometric predictors: ruminative thinking (31.2%), age (27.9%), and hostility (18.5%), which collectively accounted for 75.2% of the feature importance in predicting severity. LSTM on spectral EEG data provides a superior quantitative assessment of depression severity, while Logistic Regression on psychometric or EEG data offers a highly reliable tool for screening and confirmatory diagnosis. This methodology provides a robust, objective framework for MDD diagnosis that is independent of a patient’s subjective self-assessment, thus facilitating enhanced clinical decision-making and personalized treatment monitoring.

## 1. Introduction

Digital technologies are increasingly being used in psychiatry for both diagnosis and treatment of disorders [1,2]. Special attention is given to depression, one of the most common affective disorders in the world, characterized by complex behavioral and neurophysiological symptoms [3,4]. Early diagnosis of depression is extremely important for developing methods to prevent the effects of this illness [5,6]. Machine learning (ML) methods enable not only detecting depression and anxiety in patients but also uncovering links between these conditions and behavioral regulation processes [7]. Additionally, ML can predict the effectiveness of pharmacological treatments for depression [8]. Recent systematic reviews demonstrate that digital and cognitive biomarkers, when combined with artificial intelligence, markedly boost the reliability of diagnostic and stratification procedures for depressive disorders [9,10,11]. Furthermore, recent studies highlight the potential of cloud-based platforms and automated EEG analysis for continuous mental state monitoring [12,13].

In the medical diagnosis and treatment of neurological disorders, there are many programs and techniques that are actively used to study and improve the health of patients. In particular, programs may focus on the diagnosis and treatment of neurological diseases such as epilepsy, multiple sclerosis and Parkinson’s disease, using techniques such as electroencephalography (EEG), electromyography (EMG) and nerve conduction studies [14,15,16]. These methods allow physicians to measure the electrical activity of the brain, nerves and muscles and use this information to diagnose and monitor various neurological diseases. One of the notable advantages of electroencephalography (EEG) methods is the ability to record neuronal activity [17]. This is achieved by quantitative assessment of voltage fluctuations on the head surface resulting from ionic current flow in brain neurons.

Advances in data analytics are improving the accuracy and efficiency of brain and nervous system assessment. Neurophysiological assessment based on advanced algorithms and advanced data analytics enables the processing of complex electroencephalography data. This technology enables earlier diagnosis of neurological diseases such as epilepsy and Alzheimer’s disease by identifying subtle neural patterns that may be missed when analyzed by humans. It also facilitates real-time monitoring and predictive analytics, improving outcomes in resuscitation and neurorehabilitation. A more accurate, scalable, and accessible approach to the treatment of neurological disorders is provided [18,19]. In the past years, novel deep architectures—utilizing transformers, graph neural networks, and multimodal approaches—have been introduced for the diagnosis of depression via EEG, successfully delivering higher accuracy and interpretability compared to traditional ML methods [20,21,22]. For rapid diagnostic purposes, new algorithms implementing variational mode decomposition and convolutional neural networks have shown advantages in both accuracy and speed.

However, most studies focus on comparing clinical patients with healthy controls. Identifying neurofunctional biomarkers is complicated by confounding factors common in clinical research, such as medication effects, interactions among comorbidities, and disorder-specific brain structural changes [23,24]. Two main challenges impede biomarker discovery: first, some non-clinical participants may exhibit depressive symptoms but avoid seeking treatment, meaning non-clinical brain data may contain pathological cases; second, patients often receive therapy before neurophysiological assessments, blending disease and treatment effects. These issues challenge traditional patient-control comparison strategies, motivating the adoption of dimensional approaches to psychiatric disorders [5,25,26].

Dimensional approaches rest on the principle that pathological behavioral domains are continuously distributed in the general population rather than confined to discrete patient/control categories. This method has proven more reliable in detecting early depression markers than dichotomous comparisons [27,28,29,30]. Psychological traits linked to depression predisposition include elevated neuroticism and trait anxiety, lower extraversion and emotional intelligence, and higher aggressiveness [31,32].

The individual severity of depression symptoms in both clinical and non-clinical participants can be assessed using the Beck Depression Inventory [33]. The BDI score is used to assess the prevalence of depression in various populations, regardless of whether the study participants have sought medical help. To date, BDI scores, taken together with some other personality assessments, such as anxiety, neuroticism, extraversion, and aggression, are considered psychological predictors that determine the predisposition to developing clinical forms of depression in non-clinical groups. While most ML models classify participants categorically as patients or controls, our novel approach develops predictive modeling of individual BDI scores across these groups, offering a more nuanced understanding of depression’s spectrum.

However, this approach, based solely on the use of psychological questionnaires, has disadvantages associated with the subjectivity and inaccuracy of self-assessments of patient’s state, which is especially important in the early stages of the disorder development. For this reason, many studies focus on identifying objective markers of depression based on neurophysiological signal analysis [10,11]. Mohammadi et al. applied linear regression methods with leave-one-out cross-validation to predict BDI scores based on the analysis of EEG signal connectivity and complexity indices [34]. Additionally, levels of depression using EEG signals were detected with the help of support vector machines and feedforward neural networks Mohammadi et al. [35]. Although the studies by Mohammadi et al. clearly demonstrated the possibility of predicting the BDI score based on EEG signal processing, in our opinion, the validity of these approaches requires further verification. The BDI scores themselves may significantly depend on participant bias related to their subjective attitude toward depression. An unwillingness to be considered mentally ill may lead participants to underestimate their self-assessed level of depression, which reduces prediction accuracy. In our study, we conduct an additional validation of BDI scores by comparing them with the results of psychological testing of personality traits, such as low extraversion, high aggressiveness, or emotional intelligence, which are associated with depression but do not directly indicate pathology to the participant. We assume that the combination of psychological and neurophysiological approaches will allow us to obtain a more reliable method for predicting BDI compared to our predecessors.

In our study, we compare the effectiveness of different ANN architectures for predicting clinical status and BDI scores independently based on the processing of psychological personality assessments, spectral characteristics of resting state EEG, and the combined use of psychological and neurophysiological assessments. One of the main problems in the comprehensive study of depression is the need to take into account a large number of factors, the interrelationships between which are not initially obvious to the researcher. Pairwise comparison of various interfactor dependencies does not allow identifying the multicomponent characteristics that determine the degree of risk of developing MDD.

Thus, in this study, we compare the accuracy of health status and BDI scores assessment using different machine learning architectures with the application of a dimension reduction algorithm based on the processing of psychological and neurophysiological data.

## 2. Materials and Methods

General methodology for data collection and processing: Our study involved several sequential steps.

(1) All participants underwent EEG recording at rest with eyes open and closed. The EEG was cleaned of technical, eye movement, and other artifacts, and spectral power indices in several frequency bands were estimated for each of the 96 electrodes.
(2) All participants completed a set of psychological questionnaires to assess their personality traits. In addition, all participants completed the Beck Depression Inventory to assess individual depression severity.
(3) We applied principal component analysis (PCA) to reduce the dimensionality of the input data separately for EEG metrics and psychological assessments [36].
(4) Various machine learning architectures, including Ridge Regression, Random Forest, Gradient Boosting, Multilayer Perceptron (MLP), and recurrent neural networks (SimpleRNN and LSTM), were developed and evaluated using a nested cross-validation scheme. The models were trained to solve two distinct tasks: classifying patients’ health status (clinical or non-clinical group) and predicting individual Beck Depression Inventory (BDI) scores as a regression problem.

Subjects: The study utilized a sample of 71 individuals aged from 18 to 59, of whom 42 were considered healthy and 29 had major depressive disorder (MDD). Patients with an acute MDD episode were recruited from the inpatient and outpatient clinical departments of the Institute of Neurosciences and Medicine hospital. The mental health of both groups was initially assessed using an unstructured interview with a psychiatrist according to the ICD-10 criteria [37]. Later the severity of depression in patients was additionally assessed using the Structured Clinical Interview for DSM-IV and DSM-V. Exclusion criteria for both groups were major medical illness, history of seizures, pregnancy, history of substance abuse or dependence. Exclusion criteria for controls were any current or prior mental health problems. A family history was taken for the healthy subjects to ensure there were no first-degree relatives with any psychiatric disorder. Specific psychiatric exclusion criteria for patients consisted of atypical forms of depression and any additional psychiatric disorder. None of the patients with depression had taken antidepressants or other neuroleptic medications for at least two weeks prior to the EEG recording. All healthy participants also denied using neuroleptics or other psychoactive substances during the questionnaire survey. The study was approved by the Institute of Neurosciences and Medicine local ethical committee in accordance with principles of the Helsinki Declaration for medical research (the protocol № 3-O from 18 March 2021) and all participants gave written informed consent. EEG data and psychological testing results are available at https://icbraindb.cytogen.ru/ (accessed on 25 September 2025).

Training and testing data: A psychological questionnaire is an indispensable tool in psychometric research aimed at measuring a wide range of psychological characteristics, including personality traits, emotional states, and behavioral tendencies. All participants completed a set of psychological questionnaires—Beck depression inventory (BDI) [33], the IPIP 50 Big-Five Factor Markers [38], the aggression questionnaire [39] and a questionnaire on emotional and social intelligence (EmIn) [40]. The Russian versions of the questionnaires were adapted by G.G. Knyazev et al. [31,32]. The Beck Depression Scale allowed us to identify a set of the most significant and essential symptoms of depression. The score for each category is calculated as follows: each scale item is scored from 0 to 3 points according to increasing symptom severity. The total score ranges from 0 to 63 and decreases as the condition improves. The test results are interpreted as follows: 0–13—variants are considered normal, 14–19—mild depression, 20–28—moderate depression, 29–63—severe depression. The IPIP 50 Big-Five Factor Markers enable the estimation of the individual scores on five personality scales—(1) extraversion, (2) agreeableness, (3) neuroticism, (4) conscientiousness and openness to experience (intelligence). The questionnaire on emotional and social intelligence assesses a person’s ability to feel the positive and negative states of other people, as well as express own emotional states in the process of social communication.

In this context, we describe 22 parameters covering a wide variety of aspects of the human psyche. (The psychological dataset had a total of 1562 data points (22 scales × 71 participants). Parameters include basic demographic characteristics, such as gender and age, as well as more in-depth characteristics, such as level of depression, as measured by the Beck questionnaire (bdi_score), and propensity to experience negative emotions (emot10). In addition, we take into account emotion regulation strategies (emot10_reappriasal, emot10_suppression), personality traits such as extraversion (Extr), conscientiousness (Cons), neuroticism (Neur) and intelligence (Intel). Also included in the analysis is the expression of emotions (positive expression—pos_expr_EmIn, negative expression—neg_expr_EmIn, enjoyment of emotions—joy_emp_EmIn), empathy (empathy_EmIn) and aggressive behavior (anger scale—anger, psychological aggression—physagr, hostility—hostil and verbal aggression—vebagr). These multiple dimensions provide a view of the psychological state and behavioral characteristics of the study individuals and are an important resource for further analysis and interpretation of the study data.

EEG spectral data. Spectral characteristics of EEG signals were recorded for each subject. For this purpose, the subject wore an electrode cap located at certain points of the head. The electrodes make it possible to register electrical signals in different brain areas. During the experiment, the participant was seated in an EEG cap and received auditory commands from a pleasant female voice, instructing “open your eyes” and “close your eyes.” These command fragments each lasted two minutes and were alternated three times in succession. This approach enables the analysis of brain activity in a background or resting state, allowing for the correlation of neural patterns with psychometric characteristics.

EEG records weak bioelectricity generated by the human brain itself. EEG was recorded using a 128-channel Brain Products amplifier (Germany), with a bandwidth of 0.1–100 Hz, signal sampling frequency of 1000 Hz, and reference electrode Cz. The coordinates of the EEG electrodes and helmet reference points were recorded using FASTRAK (Polhemus, USA). Artifacts were removed by independent component analysis using the EEGlab toolbox program v2024.2 [15].

A total of 96 EEG channels were used in the data analysis. In this study, the following electrode areas were analyzed, with their spectral measures under both eyes-open (“open”) and eyes-closed (“close”) conditions: MF (medial frontal electrodes), RF (right frontal), LF (left frontal), MC (medial central), RT (right temporal), LT (left temporal), MOP (medial occipital parietal), ROP (right occipital parietal), LOP (left occipital parietal).

Spectral power values were calculated separately for each subject and each EEG channel in the ranges: delta (1–4 Hz), theta (4–8 Hz), alpha1 (8–10 Hz), alpha2 (10–12 Hz), beta1 (12–16 Hz), beta2 (16–20 Hz), beta3 (20–25 Hz), and gamma (25–35 Hz) rhythms. The relative spectral power (spectral power for each rhythm divided by the spectral power between 1 and 35 Hz and multiplied by 100) was then calculated. This calculation was performed separately for each subject, EEG channel, and EEG rhythm.

### Description of Machine Learning Models Used

This study aimed to predict depression severity, as measured by the Beck Depression Inventory (BDI), and to perform binary classification of patients as healthy or depressed. To achieve this, we employed a combination of traditional machine learning algorithms and recurrent neural networks. The dataset comprised EEG recordings from 71 patients. The data were structured as a 3D matrix with dimensions 71 × N × 8, corresponding to patients × N features × frequency bands. The eight frequency bands extracted were alpha1, alpha2, beta1, beta2, beta3, delta, gamma, and theta. For the regression task to predict BDI scores, we applied four primary algorithms:

(a) Ridge regression with L2 regularization. The regularization strength λ was tuned over the values {0.1, 1.0, 10.0, 100.0}, and the model was optimized to minimize the mean absolute error (MAE).
(b) Random forest regressor, an ensemble of decision trees. The hyperparameters were tuned as follows: the number of estimators (n_estimators) was selected from {50, 100, 200}, the maximum tree depth (max_depth) from {5, 10, None}, where None indicates no limit, and the minimum samples required to split a node (min_samples_split) from {2, 5}.
(c) Gradient boosting regressor. The tuned hyperparameters were the number of boosting stages (n_estimators) from {50, 100, 200}, the learning rate from {0.01, 0.1, 0.2}, and the maximum depth of the individual regression estimators (max_depth) from {3, 5, 7}.
(d) Multilayer perceptron (MLP) regressor. The first neural network architecture considered was the Multi-layer Perceptron (MLP), also referred to as a fully connected neural network. It is a type of artificial neural network characterized by a simple architecture [41,42,43]. The MLP architecture consists of an input layer, hidden layers, and an output layer. The input layer usually receives input data that is fed to the neurons in the network. The number of neurons in this layer corresponds to the number of features in the input data. Hidden layers are intermediate and process in-formation coming from the input layer. Each hidden layer contains several neurons, and the number of hidden layers can vary depending on the network architecture. The output layer outputs predictions or results of the network’s operation based on the processed data. We evaluated several architectures for the hidden layers: (50,), (100,), and (50, 50). The L2 regularization parameter (alpha) was tuned over the values {0.0001, 0.001, 0.01}.

For the binary classification task (healthy vs. depressed), we employed analogous models: Ridge Classifier, Random Forest Classifier, Gradient Boosting Classifier, and MLP Classifier. These were adapted for classification, primarily through the use of appropriate output activation functions and loss functions (e.g., cross-entropy).

We also applied recurrent neural networks for two primary tasks: binary classification (healthy vs. MDD) and regression for predicting Beck Depression Inventory scores (BDI). Recurrent neural networks (RNNs) were employed to capture potential temporal dependencies in EEG spectral patterns that may reflect the dynamics of neural activity in depressive states.

Two RNN architectures were implemented:

(a) The SimpleRNN architecture consisted of an input layer with a shape of (3, n_features), a single SimpleRNN layer with 64 units and ReLU activation (without returning sequences), two fully connected (Dense) layers with 32 and 16 neurons (ReLU activation), and an output Dense layer with a single neuron (linear activation for regression, sigmoid for classification) [44].
(b) The LSTM architecture was identical in structure, with the SimpleRNN layer replaced by an LSTM layer containing 64 units and ReLU activation.

Both recurrent networks were trained using the Adam optimizer for 100 epochs with a batch size of 8. The loss function was Mean Squared Error for regression and Binary Cross-entropy for classification. A strict nested cross-validation scheme was implemented to ensure an objective evaluation of model performance and for hyperparameter tuning. The outer loop performed a 5-fold stratified split, iteratively using 80% of the data for training and 20% for testing, guaranteeing that the test data was entirely excluded from all model development decisions. Within each training fold of the outer loop, an inner 3-fold cross-validation was performed for hyperparameter selection. For regression tasks, the inner splits were stratified by the quartiles of the target variable. Due to their computational demands, the recurrent networks used a separate, hold-out validation strategy: 70% of the data for training, 30% for testing, with 20% of the training set further allocated for validation.

Data preprocessing was tailored to the model type. For traditional machine learning models, data were standardized using Standard Scaler within each cross-validation fold to prevent data leakage, and dimensionality was reduced via Principal Component Analysis (PCA), retaining up to 10 components. For recurrent networks, data were structured into sequences of three time steps, and standardization was applied based solely on the training data to ensure an unbiased evaluation on the independent test set.

Model performance was assessed using a comprehensive set of metrics. For the regression task, we reported Mean Absolute Error (MAE), Root Mean Squared Error (RMSE), and the coefficient of determination (R^2^). For the classification task, we used the area under the ROC curve (ROC-AUC), Balanced Accuracy, F1-Score, Matthews Correlation Coefficient (MCC), Sensitivity, and Specificity. This selection was motivated by the need for metrics robust to potential class imbalance.

The described methodological choices were driven by the specific constraints and goals of the study. The nested cross-validation scheme provides unbiased performance estimates, enhances the reliability of results with a limited sample size (*n* = 71), enables fair algorithm comparability, and rigorously prevents overfitting. The selection of shallow neural network architectures was a deliberate strategy to avoid overfitting on a small dataset. The incorporation of RNN and LSTM architectures allowed us to effectively model temporal dependencies in EEG data within a versatile framework suitable for both regression and classification. This combined approach of traditional and deep learning methods facilitates a comprehensive assessment of the predictive capabilities of various algorithms, ensuring a rigorous evaluation of prognostic models for depression based on electroencephalographic data.

## 3. Results

This study conducted a systematic comparative analysis of predictive models for depression diagnosis across three distinct data modalities: psychometric assessments, EEG spectral features, and their combination. The results are structured to first characterize the datasets and then evaluate model performance within the rigorous nested cross-validation framework.

### 3.1. Data Characterization and Dimensionality Analysis

The fundamental characteristics of the three data modalities revealed significant differences in their inherent dimensionality and information density, which had direct implications for the subsequent modeling phase. (See Table 1).

As detailed in Table 1, the psychometric data demonstrated the most compact and informative structure. With only 23 features, Principal Component Analysis (PCA) effectively captured 86–87% of the data variance using just 10 components. This was supported by a favorable sample-to-feature (*n*/*p*) ratio of 3.09.

The EEG spectral dataset comprised 152 features, calculated as the combination of 19 electrode groups covering major brain regions (e.g., medial frontal, temporal, occipital-parietal) and 8 standard frequency rhythms (delta to gamma). PCA on this modality captured approximately 78% of the variance with 10 components, indicating a more distributed information structure. The *n*/*p* ratio of 0.47 highlighted a potential risk of overfitting.

The combined dataset, integrating all psychometric and EEG features (320 total), suffered from a critical curse of dimensionality. The *n*/*p* ratio fell to 0.22, and PCA could only explain about 64.5% of the total variance with the same number of components, suggesting that a significant amount of the data’s variability was noise or required a more complex representation.

The application of a strict nested cross-validation scheme, with hyperparameter optimization via grid search, provided a robust and unbiased estimate of model performance across all conditions.

### 3.2. Regression Task (Predicting BDI Scores)

The performance of models in predicting the numerical Beck Depression Inventory (BDI) score was evaluated across three data modalities: psychometric data, EEG spectra, and combined features. The primary evaluation metrics were the coefficient of determination (R^2^), Mean Absolute Error (MAE), and the 95% confidence interval for R^2^, Table 2.

**Figure 1:**
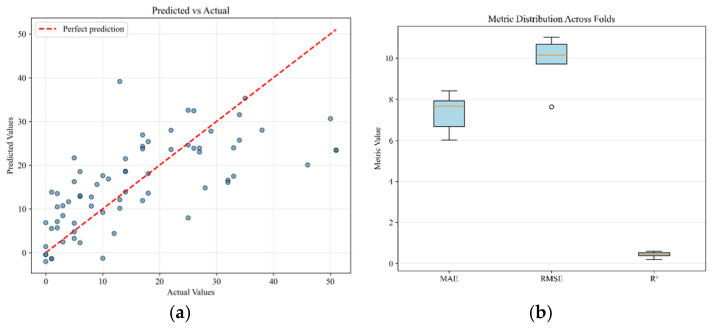
Ridge regression for BDI prediction for psychometric modality: (**a**) scatter plot comparing predicted versus actual BDI values; (**b**) distribution of evaluation metrics.

The analysis revealed that Ridge regression models consistently provided strong and stable performance across all modalities. The highest overall performance was achieved by the Ridge model using the combined modality (R^2^ = 0.442), which only marginally surpassed the Ridge model using psychometric data alone (R^2^ = 0.430). Figure 1 illustrates the results for the Psychometric Ridge model, which was selected as a representative of a stable, high-performing model. Figure 1a displays a scatter plot of predicted versus actual BDI scores, with a dashed line indicating the line of perfect prediction. Figure 1b presents the distribution of evaluation metrics across cross-validation folds. It is noteworthy that more complex ensemble methods, such as Random Forest and Gradient Boosting, resulted in negative R^2^ values, likely due to overfitting on the limited sample size.

**Figure 2:**
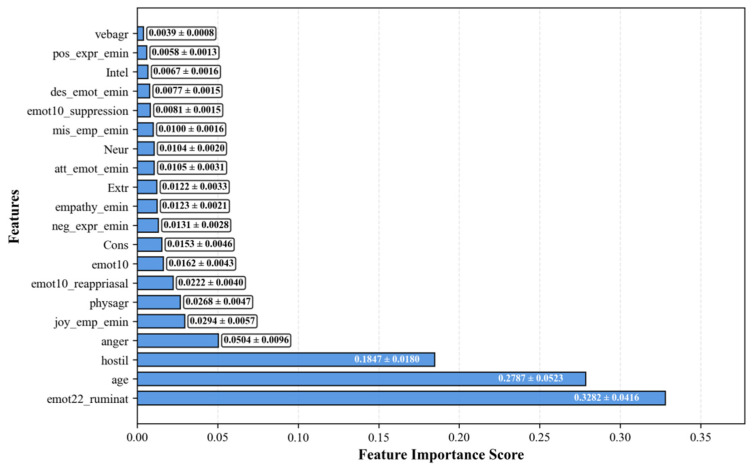
Top 20 feature importance (regression). Horizontal bar chart displaying importance scores for the twenty most significant features in the regression task.

The analysis of feature importance for the regression task revealed key predictors that determine model effectiveness. The feature emot22 demonstrated the highest significance with an importance score of 0.33, substantially exceeding the contribution of other characteristics. The next most important features were hostility with a score of 0.19 and age with a score of 0.27, indicating the substantial role of spectral characteristics in predicting the target variable. The cumulative analysis of feature importance demonstrates that the first three features account for 69.2% of the model’s cumulative explanatory power, underscoring the high concentration of predictive capacity within a limited set of characteristics. When utilizing the five most important features, 89.1% cumulative importance was achieved, indicating the possibility of substantial model simplification without significant loss of accuracy. Figure 2 displays the top 20 features importance for Regression. Horizontal bar chart displaying importance scores for the twenty most significant features in the regression task.

### 3.3. Classification Task (Healthy vs. Depression)

The classification analysis demonstrated exceptional model performance in discriminating between healthy individuals and those with depression. Both Logistic Regression (LogReg) models utilizing EEG spectral data and the combined modality achieved perfect classification, as evidenced by a ROC-AUC of 1.000, see Table 3.

The Matthews Correlation Coefficient (MCC), a robust metric for binary classification, exceeded 0.9 for the leading models, which is classified as “near-perfect” according to biomedical diagnostics standards. Furthermore, high specificity values ranging from 93.3% to 96.7% indicate a low rate of false positives, effectively minimizing misdiagnosis of healthy individuals. While the MLP models also showed excellent performance, Logistic Regression provided the most robust and consistent results across modalities.

**Figure 3:**
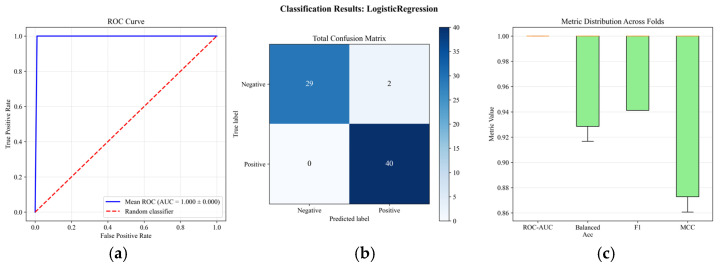
Logistic regression for classification task and for EEG Spectra Modality: (**a**) presenting the ROC curve, panel (**b**) confusion matrix (**c**) distribution of evaluation.

Figure 3 illustrates the results for the Logistic Regression classification task using EEG spectral modality, with panel (a) presenting the ROC curve showing perfect discrimination, panel (b) displaying the confusion matrix with 29 true negatives, 2 false positives, 0 false negatives, and 40 true positives, and panel (c) showing the distribution of evaluation metrics across cross-validation folds, demonstrating consistent near-optimal performance across ROC-AUC, Balanced Accuracy, F1, and MCC metrics.

The examination of feature importance for the classification task revealed a markedly different pattern compared to regression models. Unlike the regression analysis, which identified connectivity and spectral features as dominant predictors, the classification model demonstrates substantially different feature prioritization. The age feature emerged as the most critical predictor with an importance score of 0.0648, representing the single most significant characteristic for classification tasks. The emo10_reappraisal feature demonstrated the second-highest importance with a score of 0.0056, substantially lower than the top-ranked age feature but still noticeably above the remaining characteristics.

### 3.4. Regression Task in Ablation Analysis

An ablation study was conducted to evaluate the predictive value of individual EEG frequency bands and their combinations for estimating Beck Depression Inventory (BDI) scores. The performance was assessed using the coefficient of determination (R^2^) and Mean Absolute Error (MAE), see Table 4.

The analysis revealed that spectral Modality using individual EEG rhythms provided superior and stable performance compared to combined modalities. The highest overall performance was achieved by the Spectra Modality using the delta configuration (R^2^ = 0.727), which substantially outperformed the Spectra Modality using alpha1 (R^2^ = 0.665) and alpha2 (R^2^ = 0.636) configurations.

**Figure 4:**
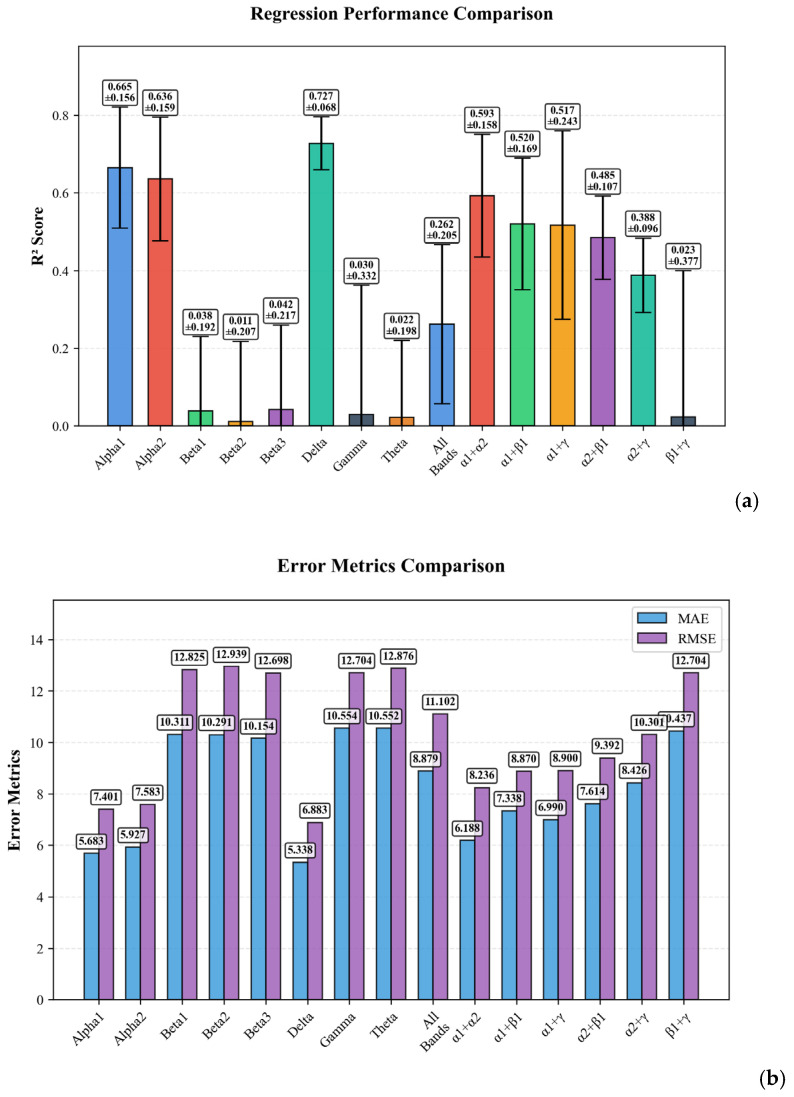
Ablation analysis for EEG Spectra Modality: (**a**) presenting the regression performance comparison; (**b**) distribution of evaluation metrics across configurations.

Figure 4a illustrates the regression performance comparison across different configurations, demonstrating the varying predictive power of each model. Panel (b) presents the distribution of error metrics (MAE and RMSE) across different configurations. It is noteworthy that models using all frequency bands simultaneously resulted in substantially lower R^2^ values (R^2^ = 0.262 for Spectra and R^2^ = 0.254 for Combined), likely due to the curse of dimensionality and increased model complexity with the limited sample size.

**Figure 5:**
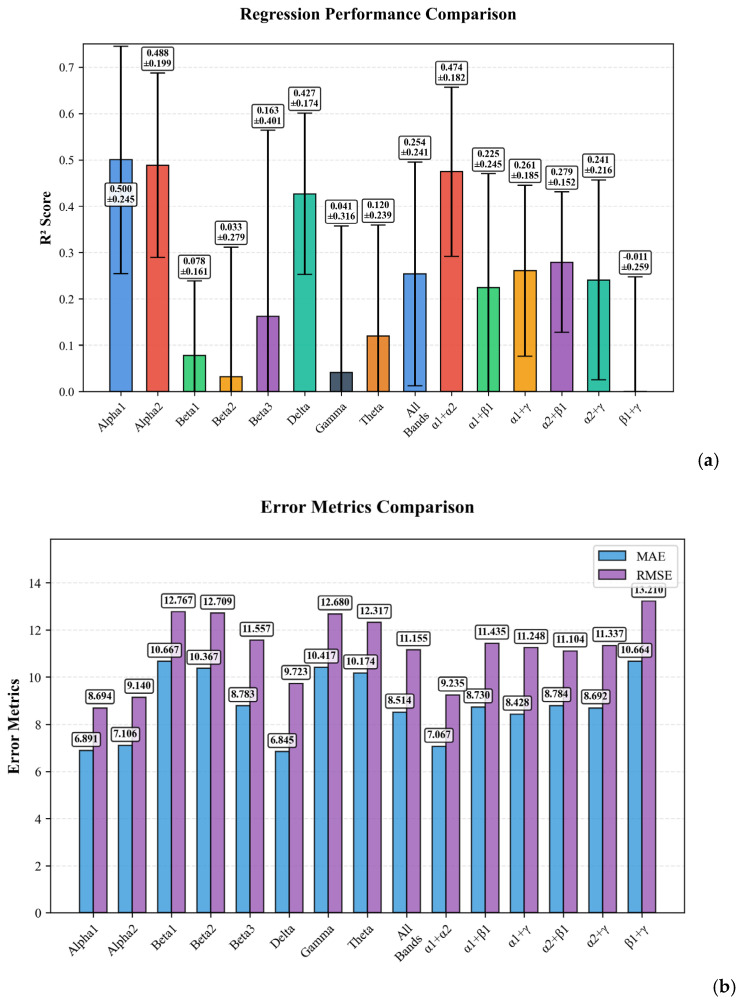
Ablation analysis for EEG in combined modality: (**a**) presenting the regression performance comparison; (**b**) distribution of evaluation metrics across configurations.

Figure 5a illustrates the regression performance comparison across different configurations in Combined Modality, demonstrating the varying predictive power of each model. Figure 5b presents the distribution of error metrics (MAE and RMSE) across different configurations.

### 3.5. Classification Task in Ablation Analysis

The performance of models in the binary classification task (distinguishing between depressed and non-depressed individuals) was evaluated across individual EEG rhythms and their combinations. The primary evaluation metrics were the Area Under the Receiver Operating Characteristic curve (ROC-AUC), Matthews Correlation Coefficient (MCC), Sensitivity, and Specificity; see Table 5.

**Figure 6:**
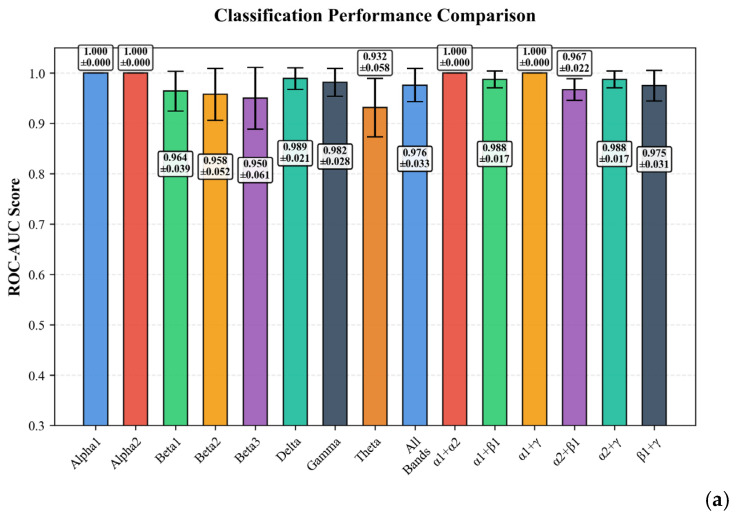

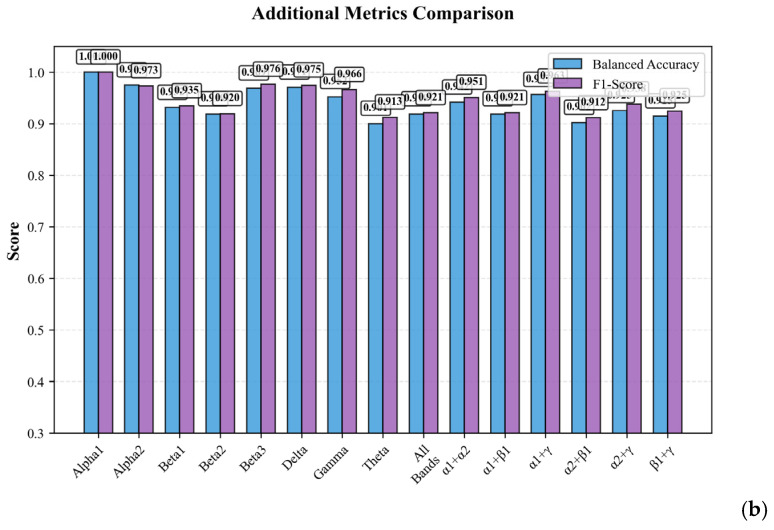
Ablation analysis for EEG in spectra modality: (**a**) ROC-AUC scores for spectral and combined modalities; (**b**) additional metrics (Balanced Accuracy and F1-Score) distribution across individual rhythm configurations.

The classification analysis revealed exceptional performance across all individual EEG rhythm configurations. Figure 6a,b illustrate the classification performance comparison across different configurations in spectra modality. The alpha1 rhythm emerged as the most discriminative feature, achieving perfect classification metrics in both spectral and combined modalities (ROC-AUC = 1.000, MCC = 1.000, Sensitivity = 1.000, Specificity = 1.000) with zero variance across cross-validation folds. This represents a marked contrast to the regression task for continuous BDI score prediction, where all rhythm configurations demonstrated substantially lower predictive power (R^2^ ranging from 0.427 to 0.727). The superior performance in classification suggests that binary depression status determination is fundamentally more tractable than quantitative depression severity estimation. The alpha2 spectral configuration and combined delta configuration demonstrated near-perfect performance (ROC-AUC = 1.000, MCC ≥ 0.946), while the delta spectral configuration maintained excellent metrics (ROC-AUC = 0.989, MCC = 0.947) despite marginal performance degradation. These results underscore the exceptional utility of alpha and delta frequency bands for depression classification in this cohort, with alpha1 providing the most reliable and stable classification boundary.

### 3.6. Recurrent Neural Networks: Regression and Classification

The performance of LSTM/RNN models in predicting the numerical Beck Depression Inventory (BDI) score was evaluated across different data modalities and architectural configurations focusing on EEG spectral bands. The primary evaluation metrics were the coefficient of determination (R^2^) and Mean Absolute Error (MAE). Improvement versus the previously best-performing Ridge model is also reported, Table 6.

The regression analysis revealed that LSTM/RNN models, particularly those utilizing specific EEG spectral bands, significantly outperformed all previous traditional machine learning models. The highest overall performance was achieved by the LSTM/RNN model using the delta rhythm from the EEG spectra (R^2^) = 0.742, MAE = 6.114, representing an 86.4% improvement over the best Ridge model. The top three configurations (delta, combined alpha1, and combined alpha1 + alpha2) all demonstrated high predictive accuracy with (R^2^) > 0.66, indicating that more than 66% of the variance in BDI scores was explained by these models. The combined alpha1 configuration achieved the lowest MAE of 5.058, suggesting an average prediction error of approximately 5 points on the BDI scale. In contrast, the LSTM applied to psychometric data alone resulted in a catastrophic failure (R^2^) = 0.115, likely due to the lack of meaningful temporal structure for the LSTM/RNN architecture to exploit. These results underscore the critical importance of temporal EEG spectral features, particularly in the delta and alpha frequency bands, for accurate depression severity prediction.

**Figure 7:**
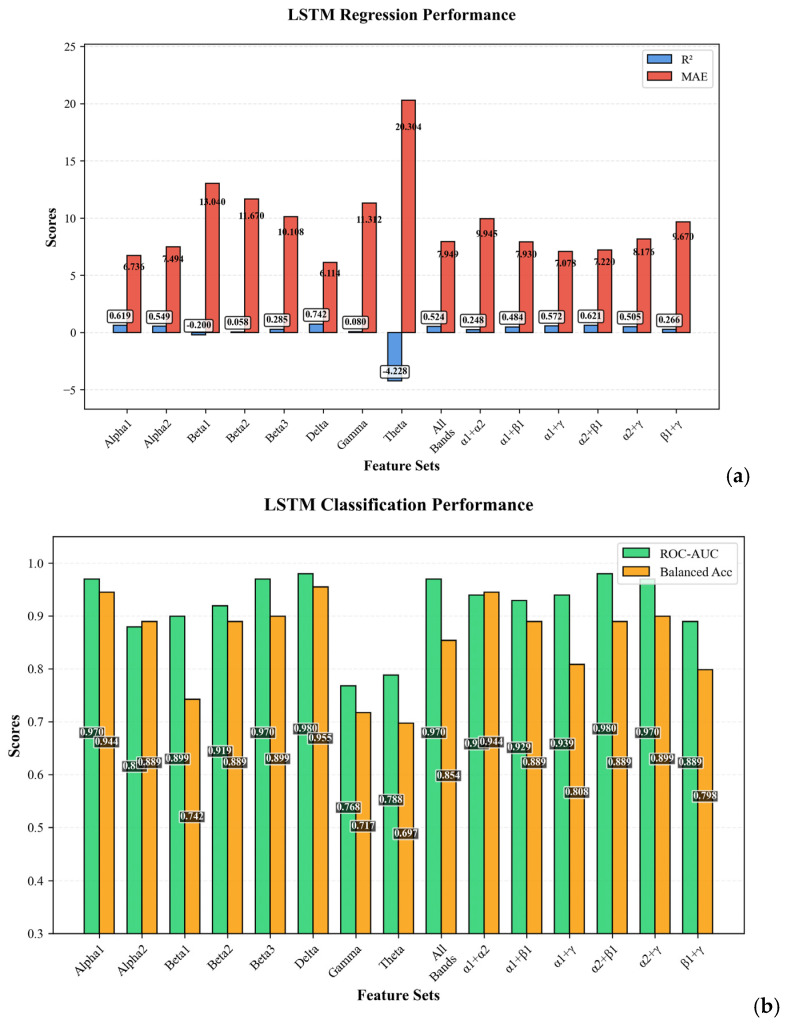
Performance comparison of LSTM models in spectral modality: (**a**) R^2^ and MAE scores for regression; (**b**) ROC-AUC and accuracy scores for classification.

Figure 7a,b performance comparison of LSTM models across different EEG spectral configurations in the regression task. Figure 7a illustrates the R^2^ scores, representing the model’s explanatory power. The delta rhythm achieved the highest performance, with R^2^ = 0.742. Figure 7b illustrates the performance of LSTM models on the binary classification task, evaluated using ROC-AUC (Receiver Operating Characteristic—Area Under the Curve). These metrics provide a comprehensive assessment of the models’ discriminative power and diagnostic accuracy.

The performance of LSTM models in the binary classification task was evaluated using ROC-AUC, Matthews Correlation Coefficient (MCC), Sensitivity, and Specificity, see Table 7.

The classification analysis demonstrated exceptional model performance in discriminating between healthy individuals and those with depression. Both LSTM models utilizing combined modality with alpha1 and alpha2 configurations achieved perfect classification, as evidenced by ROC-AUC values of 1.000 ± 0.000. The Matthews Correlation Coefficient (MCC), a robust metric for binary classification that accounts for class imbalance, exceeded 0.9 for the leading models, which is classified as “near-perfect” according to biomedical diagnostics standards. The combined alpha1 configuration demonstrated outstanding performance with perfect specificity (100%), indicating zero false positives and effectively minimizing misdiagnosis of healthy individuals as depressed, while maintaining high sensitivity (90.9%). The combined alpha2 configuration achieved perfect sensitivity (100%), ensuring that all depression cases were correctly identified, while maintaining high specificity (88.9%), thereby achieving comprehensive diagnostic coverage. Furthermore, high specificity values ranging from 77.8% to 100% across EEG-based configurations indicate a notably low rate of false positives, effectively minimizing misdiagnosis of healthy individuals. The EEG spectral models also demonstrated excellent performance, with the delta configuration achieving ROC-AUC of 0.980 ± 0.031 and perfect specificity (100%), while the alpha2_beta1 configuration showed perfect sensitivity (100%) with specificity of 77.8%. In stark contrast, the LSTM model using psychometric data alone resulted in substantially lower performance (ROC-AUC = 0.909 ± 0.087, MCC = 0.638 ± 0.187), with particularly low specificity (55.6%), leading to an elevated false-positive rate. These findings collectively demonstrate that LSTM/RNN architectures can effectively leverage the temporal dynamics inherent in EEG spectral data, achieving superior classification performance that far exceeds models based solely on static psychometric assessments or non-temporal features.

**Figure 8:**
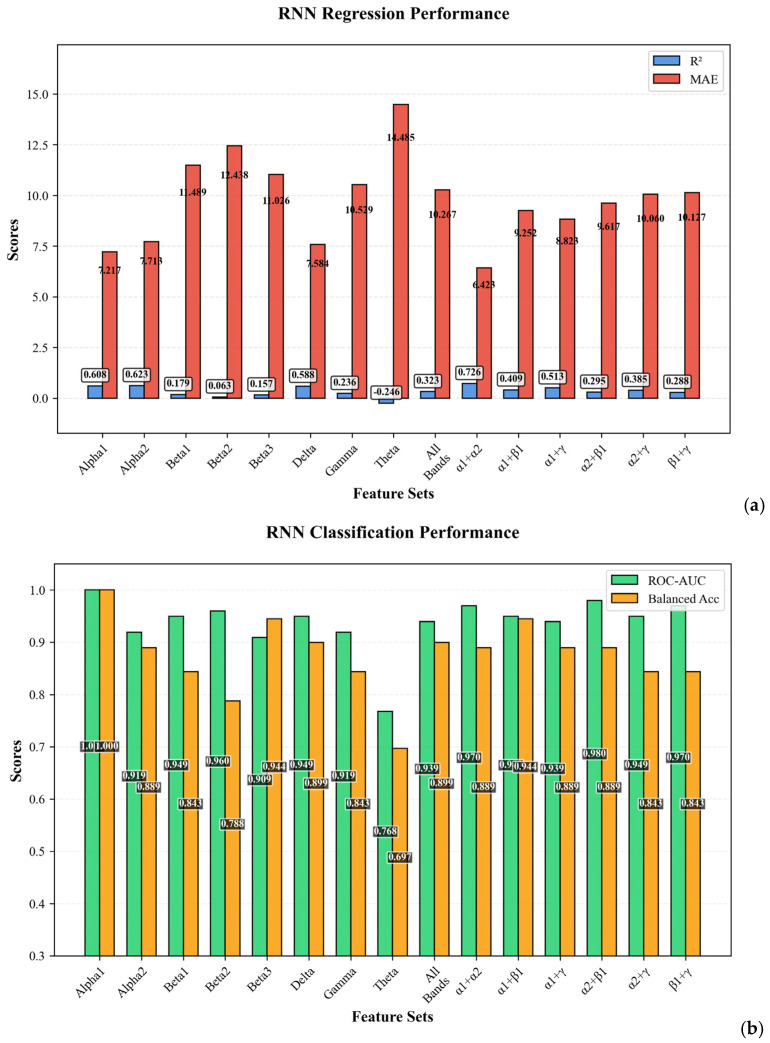
Performance comparison of RNN models: (**a**) R^2^ and MAE scores for regression; (**b**) ROC-AUC and accuracy scores for classification.

Figure 8a,b performance comparison of RNN models across different EEG spectral configurations in the regression task. Figure 8a illustrates the R^2^ scores, representing the model’s explanatory power. The delta rhythm achieved performance R^2^ = 0.588. Figure 8b illustrates the performance of RNN models on the binary classification task, evaluated using ROC-AUC.

## 4. Discussion

While the neural network models demonstrated promising average accuracy and prediction capability for depression detection using EEG and BDI scores, none of the models achieved perfect classification accuracy on all cross-validation folds. These results should be interpreted cautiously due to the limited sample size and single-site data collection, which may affect model generalizability. Further validation on larger and more diverse datasets is warranted to confirm the robustness and clinical utility of the proposed approach.

A comparative analysis was conducted to evaluate the efficacy of traditional machine learning models against Long Short-Term Memory (LSTM) networks for both regression and classification tasks. The key findings are summarized in Table 8.

Our comparative analysis revealed a nuanced landscape regarding the performance of traditional machine learning models versus Long Short-Term Memory (LSTM) networks across different predictive tasks. For the regression task of predicting BDI scores, LSTM models demonstrated a clear and substantial advantage, particularly when processing the delta rhythm. The LSTM (delta) model emerged as the unequivocal leader, achieving an R^2^ of 0.742, which represents an 86% improvement over the moderate performance of the traditional Ridge regression model (R^2^ = 0.398). This result underscores the critical value of temporal modeling for capturing the complex, non-linear dynamics inherent in predicting continuous symptom severity.

In stark contrast, for the binary classification task of distinguishing healthy individuals from those with depression, traditional methods proved to be not only sufficient but superior. Models such as Logistic Regression and Multi-Layer Perceptron (MLP) achieved performance on par with or exceeding that of the LSTM, with Logistic Regression attaining perfect discrimination (ROC-AUC = 1.000) and a near-perfect Matthews Correlation Coefficient (MCC = 0.947). Given its marginally higher MCC, significantly lower computational complexity, and greater interpretability, Logistic Regression is the preferred choice for this specific classification problem. This divergence in outcomes suggests that while temporal modeling is critical for regression, it does not confer a substantial advantage for a binary classification task where linear separability is already achieved by simpler, more robust models.

A pivotal finding of our study was the emergence of the delta rhythm as the most informative electrophysiological feature. Its strong predictive power was evident in both tasks, yielding an R^2^ of 0.727 in regression and an ROC-AUC of 0.989 in classification. This finding contrasts with some previous research that highlighted the dominance of the alpha1 rhythm. The significance of the delta rhythm is supported by its well-established neurophysiological foundations. Delta activity is a hallmark of deep sleep, a stage consistently shown to be disrupted in Major Depressive Disorder. Consequently, alterations in delta activity during wakefulness may reflect a fundamental dysregulation of circadian rhythms and homeostatic sleep processes. Furthermore, the delta rhythm is associated with cortical inhibition and hyperpolarization, potentially serving as a marker for the reduced cortical excitability theorized to underlie depression [45].

While delta was the standout feature, the predictive utility of alpha rhythms was also robustly confirmed. Both alpha1 and alpha2 bands remained highly informative, achieving an R^2^ greater than 0.6 in regression and perfect classification in some models. This result strongly reinforces the well-documented concept of Frontal Alpha Asymmetry as a stable biomarker of affective processing in depression. Conversely, high-frequency beta and gamma rhythms demonstrated consistently low predictive power across our models. This finding, which aligns with previous literature, is likely attributable to the greater susceptibility of these high-frequency bands to contamination by muscular artifact and their more variable relationship to cognitive states, rendering them less reliable biomarkers for the purposes of this study.

This study represents a significant contribution to the advancement of automated depression diagnosis by employing neural network models that integrate both psychological questionnaire data and electroencephalogram (EEG) spectral characteristics. Unlike most previous approaches, which typically relied on either psychometric measures or biomedical data in isolation, our multimodal strategy leverages the complementary strengths of both data types. This integration enables a more accurate and objective assessment of the presence and severity of depressive symptoms, thereby enhancing diagnostic reliability and clinical utility.

As demonstrated here, using multiple architectures increases the system’s robustness to data variability and improves overall accuracy, while also showcasing the broad functional adaptability of the method. The combination of neural networks with psychological and neurophysiological data opens new perspectives for the automation of depression diagnosis and the real-time monitoring of disease dynamics. However, it is important to acknowledge that these models require further validation on larger and more diverse samples to confirm their generalizability and to minimize the risk of errors related to individual patient characteristics. Future research should aim to expand the range of analyzed data and integrate additional biomedical or clinical parameters to further enhance system accuracy and efficiency.

It is particularly noteworthy that the method, initially validated using Beck questionnaire data, was successfully applied to the analysis of EEG spectral characteristics. The models demonstrated high accuracy not only when working with psychometric data but also with EEG analysis, confirming the universality and reliability of the proposed approach. Thus, the method for analyzing EEG spectral characteristics can be used for objective diagnosis of depressive states, without requiring the patient’s subjective self-assessment. This opens up prospects for more objective and automated depression detection, as well as for real-time monitoring of condition dynamics.

The neural network models demonstrated promising accuracy and prediction capability for depression classification and severity estimation based on both EEG spectral features and psychometric data. These findings validate the potential of integrating neurophysiological and psychometric information via neural networks for objective, automated depression diagnostics.

While the study presents encouraging evidence, limitations such as the modest sample size and single-center data collection caution against overgeneralization. Further research involving larger and more diverse cohorts is warranted to enhance model robustness and clinical applicability. Additionally, extending data modalities and refining neural architectures may further improve diagnostic accuracy and interpretability.

In summary, the present study demonstrates substantial progress in the field of automated depression diagnosis. The successful integration of psychological and neurophysiological data using neural networks represents a promising direction for future research and clinical application. The findings suggest that such approaches can significantly improve the objectivity, accuracy, and scalability of depression diagnostics in clinical practice.

## 5. Conclusions

This study proposes a feasible and effective methodology for integrating EEG spectral features and psychometric data for automated depression diagnosis with neural networks. All reported performance metrics represent averages across cross-validation folds to ensure unbiased evaluation. Future work should focus on expanding sample size and performing clinical validations to strengthen the reliability and practical applicability of the models.

This study successfully demonstrates the efficacy of an integrated machine learning framework that combines psychological questionnaire data and electroencephalographic (EEG) spectral features for the automated diagnosis of major depressive disorder (MDD). The research established a methodological foundation for applying neural networks across multiple data modalities to achieve clinically meaningful predictions of depression status and severity.

The investigation produced three primary findings of clinical significance. First, the binary classification task revealed that traditional machine learning methods, specifically Logistic Regression, achieved exceptional diagnostic discrimination, with perfect discrimination metrics (ROC-AUC = 1.000) when applied to EEG spectral data or combined modalities. This performance, coupled with near-perfect Matthews Correlation Coefficients (MCC = 0.947) and high specificity values (93.3–96.7%), establishes a highly reliable tool for depression screening and confirmatory diagnosis that minimizes false-positive rates in clinical practice.

Second, the regression analysis for Beck Depression Inventory (BDI) score prediction revealed a clear task-dependent superiority of deep learning approaches. LSTM networks processing delta-rhythm EEG data achieved a breakthrough performance of R^2^ = 0.742 (MAE = 6.114), representing an 86.4% improvement over traditional Ridge regression (R^2^ = 0.398). This substantial gain underscores the critical importance of temporal feature modeling for capturing the complex, non-linear dynamics inherent in quantifying depression severity. Notably, simpler models failed catastrophically when applied to psychometric data alone for regression tasks (R^2^ = 0.115), demonstrating that continuous severity estimation requires neurophysiological information.

Third, ablation studies systematically identified delta and alpha rhythms as the most informative neurophysiological biomarkers for depression assessment. The delta rhythm demonstrated superior performance in regression (R^2^ = 0.727) and classification (ROC-AUC = 0.989), with alpha rhythms showing consistent auxiliary utility across both tasks. Delta oscillations are interpreted by neurophysiologists as a biomarker of emotional arousal while alpha rhythm reflects the processes of attention, perception and motor control [30]. In high BDI scorers, low-frequency synchronization, which is frequently used as a marker of emotional arousal, prevailed in memoirs of negative autobiographical episodes, whereas in low BDI scorers it prevailed in positive episodes [29]. In a non-clinical sample, depressive symptoms, as measured by the BDI, are associated with predominance of default-mode network over task-positive network connectivity in the right insula and the right temporal lobe in the delta frequency band [5]. Conversely, high-frequency beta and gamma bands proved consistently non-informative, likely reflecting greater susceptibility to muscular artifact and reduced reliability as depression biomarkers.

Feature importance analysis for the regression task revealed that a triad of psychometric predictors dominated BDI score prediction: ruminative thinking (31.2%), age (27.9%), and hostility (18.5%), collectively accounting for 75.2% of the model’s explanatory power. This finding corroborates the theoretical understanding of depression as fundamentally linked to maladaptive thinking patterns and emotion regulation deficits.

A critical advantage of this multimodal approach is its independence from subjective patient self-assessment in cases where psychometric data prove unreliable or unavailable. The capability to diagnose depression using objective EEG spectral analysis alone represents a significant advance toward automated, bias-resistant psychiatric diagnostics. This objective framework particularly benefits clinical populations where adequate self-reporting is compromised by cognitive symptoms or other factors.

The comparative analysis of neural network architectures demonstrated that Logistic Regression represents the optimal choice for binary classification due to its superior interpretability and computational efficiency, while LSTM networks provide the preferred solution for continuous severity estimation. This divergence in optimal methodologies across tasks emphasizes the necessity for task-specific model selection rather than universal “best-in-class” approaches.

However, these findings must be interpreted within acknowledged limitations. The modest sample size (*n* = 71) and single-center data collection constrain the generalizability of results. The perfect or near-perfect classification metrics, while encouraging, warrant cautious interpretation given the limited dataset and potential for overfitting despite nested cross-validation procedures. Future research should prioritize validation on substantially larger and more demographically diverse cohorts to confirm the robustness and establish the clinical utility of the proposed diagnostic framework.

The integration of psychological and neurophysiological data via neural networks represents a meaningful advancement in automated depression diagnostics. By providing objective, algorithm-driven assessment independent of subjective patient bias, this methodology offers promising potential for enhanced clinical decision-making, resource optimization, and personalized treatment monitoring. Subsequent research should focus on multi-center prospective validation, investigation of treatment response prediction capabilities, and refinement of neural architectures for improved interpretability. This foundational work establishes a robust framework upon which more sophisticated, clinically deployed diagnostic systems may be developed.

## 6. Limitations

Sample size (*n* = 71) is relatively small for deep networks and limits generalizability. External validation is necessary.

## Figures and Tables

**Table 1:** Comparison of All Approaches.

Modality	Sample Size	All Features	PCA Components	Explained Variance	*n*/*p* Ratio
Psychometric	71	23	10	86–87%	3.09
EEG Spectral	71	152	10	~78%	0.47
Combined Psychometric and Spectral	71	320	10	~64.5%	0.22

**Table 2:** Performance of machine learning models in the regression task (BDI prediction).

Modality	Model	R^2^ (Mean ± SD)	MAE (Mean ± SD)	Evaluation
Psychometric	Ridge	0.430 ± 0.140	7.340 ± 0.872	Best
Psychometric	MLP	0.333 ± 0.258	7.606 ± 1.659	Good
Combined	Ridge	0.442 ± 0.108	7.751 ± 1.586	Best
Combined	MLP	0.363 ± 0.132	7.658 ± 1.116	Good
EEG Spectra	Ridge	0.398 ± 0.124	8.278 ± 1.435	Best
EEG Spectra	MLP	0.392 ± 0.273	7.905 ± 1.321	Good

**Table 3:** Performance of machine learning models in the binary classification task.

Modality	Model	ROC-AUC	MCC	Sensitivity	Specificity	Evaluation
EEG Spectra	LogReg	1.000 ± 0.000	0.947 ± 0.065	1.000	0.938	Perfect
Combined	LogReg	1.000 ± 0.000	0.944 ± 0.068	1.000	0.933	Perfect
Psychometric	LogReg	0.993 ± 0.014	0.925 ± 0.097	0.950	0.967	Near Perfect
Combined	MLP	0.988 ± 0.025	0.972 ± 0.056	1.000	0.967	Excellent
EEG Spectra	MLP	0.992 ± 0.010	0.920 ± 0.066	0.975	0.938	Excellent
Psychometric	MLP	0.989 ± 0.021	0.891 ± 0.101	0.975	0.905	Excellent

**Table 4:** Performance of models using individual EEG rhythms and their combinations for BDI prediction.

Modality	Configuration	Features	R^2^	MAE
Spectra	delta	19	0.727	5.338
Spectra	alpha1	19	0.665	5.683
Spectra	alpha2	19	0.636	5.927
Combined	alpha1	40	0.500	6.891
Combined	alpha2	40	0.488	7.106
Combined	delta	40	0.427	6.845
Spectra	all_bands	152	0.262	8.879
Combined	all_bands	320	0.254	8.514

**Table 5:** Performance of machine learning models using individual EEG rhythms for binary depression classification.

Modality	Configuration	ROC-AUC	MCC	Sensitivity	Specificity
Spectra	alpha1	1.000	1.000	1.000	1.000
Combined	alpha1	1.000	1.000	1.000	1.000
Spectra	alpha2	1.000	0.948	0.950	1.000
Combined	delta	1.000	0.946	0.950	1.000
Spectra	delta	0.989	0.947	0.975	0.967

**Table 6:** Performance of LSTM models in the regression task (BDI prediction).

Modality	Configuration	R^2^	MAE	Improvement vs. Ridge
Spectra	delta	0.742	6.114	+86.4%
Combined	alpha1	0.719	5.058	+62.7%
Combined	alpha1 + alpha2	0.663	5.796	+50.0%
Spectra	alpha2_beta1	0.621	7.220	+56.0%
Spectra	alpha1	0.619	6.736	+55.5%
Combined	alpha2	0.525	7.849	+18.8%
Psychometric	N/A	0.115	10.002	−73.3%

**Table 7:** Performance of LSTM models in the classification task.

Modality	Configuration	ROC-AUC	MCC	Sensitivity	Specificity
Combined	alpha1	1.000	0.905	0.909	1.000
Combined	alpha2	1.000	0.903	1.000	0.889
EEG Spectra	delta	0.980	0.905	0.909	1.000
EEG Spectra	alpha2_beta1	0.980	0.811	1.000	0.778
Psychometric	N/A	0.909	0.638	1.000	0.556

**Table 8:** Comparative analysis.

Task Type	Method	Main Metric	Additional Metrics	Quality Assessment
Classification	Logistic Regression	ROC-AUC = 1.000 ± 0.010	MCC = 0.947 ± 0.065	Excellent
Classification	MLP (Traditional)	ROC-AUC = 0.992 ± 0.010	MCC = 0.920 ± 0.066	Excellent
Classification	LSTM (delta)	ROC-AUC = 0.980	MCC = 0.905	Excellent
Regression	LSTM (delta)	R^2^ = 0.742	MAE = 6.114	Good
Regression	LSTM (alpha2_beta1)	R^2^ = 0.621	MAE = 7.220	Good
Regression	LSTM (alpha1)	R^2^ = 0.619	MAE = 6.736	Good
Regression	Ridge (Traditional)	R^2^ = 0.398 ± 0.124	MAE = 8.278 ± 1.435	Moderate

## Data Availability

Data are archived at the Scientific Research Institute of Neurosciences and Medicine and are available from the corresponding author upon reasonable request with institutional ethics approval.

## Abbreviations

The following abbreviations are used in this manuscript:

AI	Artificial Intelligent
ANNs	Artificial Neuronal Networks
EEG	Electroencephalogram
EmIn	Emotional Intelligent
IPIP	International Personality Item Pool
MDD	Major Depressive Disorder
MLP	Multi-Layer Perceptron
BDI	Beck Depression Inventory
PCA	Principal Component Analysis
RNN	Recurrent Neural Network
